# Precise Mapping of the CD95 Pre-Ligand Assembly Domain

**DOI:** 10.1371/journal.pone.0046236

**Published:** 2012-09-25

**Authors:** Valérie Edmond, Benoist Ghali, Aubin Penna, Jean-Luc Taupin, Sophie Daburon, Jean-François Moreau, Patrick Legembre

**Affiliations:** 1 Université de Rennes-1, Rennes, France; 2 Inserm U1085, IRSET, Rennes, France; 3 Université de Bordeaux-2, Bordeaux, France; 4 CNRS UMR 5164, Bordeaux, France; 5 CHU Bordeaux, Place Amélie Raba Léon, Bordeaux, France; University of Iowa, United States of America

## Abstract

Pre-association of CD95 at the plasma membrane is mandatory for efficient death receptor signaling. This homotrimerization occurs through self-association of an extracellular domain called the pre-ligand assembly domain (PLAD). Using novel molecular and cellular tools, we confirmed that CD95-PLAD is necessary to promote CD95 multimerization and plays a pivotal role in the transmission of apoptotic signals. However, while a human CD95 mutant deleted of the previously described PLAD domain (amino acids 1 to 66) fails to interact with its wild-type counterpart and trigger autonomous cell death, deletion of amino acids 1 to 42 does not prevent homo- or hetero (human/mouse)-oligomerization of CD95, and thus does not alter transmission of the apoptotic signal. Overall, these findings indicate that the region between amino acids 43 to 66 corresponds to the minimal motif involved in CD95 homotypic interaction and is necessary to convey an efficient apoptotic signal. Interfering with this PLAD may represent a new therapeutic strategy for altering CD95-induced apoptotic and non-apoptotic signals.

## Introduction

Despite their pleiotropic effects, ranging from apoptosis to cell proliferation, members of the TNF (Tumor Necrosis Factor)-receptor family share remarkably similar structures and modes of interaction and aggregation, indicating that subtle differences may account for the transmission of different signaling pathways. CD95 (also known as Fas), a death receptor in the TNF-receptor family, plays a crucial role in tumor surveillance, immune tolerance and homeostasis, as evidenced by studies in human patients affected by autoimmune lymphoproliferative syndrome (ALPS) type Ia [1]–[3]. The cognate ligand of CD95, CD95L, is a member of the TNF superfamily primarily found on the surface of immune cells, where it participates in the elimination of infected and transformed cells. CD95 is a 319 amino acid type 1 transmembrane glycoprotein containing three cysteine-rich domains (CRDs) in its extracellular region and is predicted to form pre-assembled homotrimers, with CRD2 and the upper part of CRD3 forming the region of interaction with CD95L [4]. Upon binding of CD95L or agonistic antibodies, the cytoplasmic portion of CD95 corresponding to the death domain (DD) recruits the adaptor molecule Fas-associated death domain protein (FADD) and the caspase-8 proenzyme, leading to caspase activation and apoptosis (*6–10*).

Several studies have shown that CD95 splice variants with truncated CRD2/CRD3 domains fail to interact with CD95L, although they remain capable of dominantly interfering with the CD95-mediated apoptotic signal [5]–[8]. This indicates that the first sub-domain of CRD1 encompasses a minimal homotypic interaction domain termed PLAD for pre-ligand assembly domain [7] that contributes to self-association of the receptor [7], [9].

Using novel molecular and cellular tools, we mapped the amino acids necessary for efficient transmission of the CD95-mediated apoptotic signal and demonstrated that the minimal domain necessary for CD95 homotypic interaction corresponds to a discrete region encompassed by amino acids 43 to 66.

## Materials and Methods

### Cells and Reagents

Agonistic IgM anti-human CD95 mAb clone 7C11, hamster anti-mouse CD95 mAb clone JO2 and anti-human CD95 mAb clone DX2 were purchased from BD Biosciences (Le Pont de Claix, France). Rabbit polyclonal anti-CD95 (C20) was purchased from Santa Cruz Biotechnology, Inc. (Heidelberg, Germany). Anti-HA mAb was from Eurogentec (Seraing, Belgium) and monoclonal anti-DsRed was obtained from Clontech (Saint-Germain-en-Laye, France). The metalloprotease-cleaved CD95L (CD95L) and its multi-aggregated counterpart (Ig-CD95L) were generated in our laboratory, as described previously [10], [11]. Murine pro-B BaF3 cells [12] were maintained in RPMI 1640 containing 8% fetal calf serum (FCS), 2 mM glutamine and murine IL-3 produced in CHO cells transfected with a murine IL3-encoding vector exactly as described previously [12]. CEM-IRC (IRC for *Ig-CD95L-Resistant*) cells were obtained as described previously [13] and maintained in RPMI1640 supplemented with 8% v/v heat-inactivated FCS and 2 mM L-glutamine at 37°C in a 5% CO_2_ incubator. CEM-IRC and BaF3 cells (5.10^6^ cells in 0.3 ml) were electroporated with 10 µg DNA at 200V/65 ms and 360V/10 ms, respectively, using the BTM 830 electroporation generator (BTX, Holliston, MA). To select stable clones, CEM-IRC cells were transfected and placed in a culture medium supplemented with 0.8 mg/ml neomycin. Cells were then cloned by limiting dilution and plasma membrane expression of CD95 was assessed by flow cytometry.

### Plasmids Construction

The signal peptide (SP)-encoding sequence of CD95 (amino acids 1 to 16; [14]) was amplified by PCR with a primer in which the HA sequence tag has been added to the 5′ end. This amplicon was digested by HindIII/EcoRI and inserted into a pcDNA3-(SP^CD95^HA) vector. The CD95 sequence lacking SP and encompassing full-length CD95 or CD95 devoid of amino acids 1–42 or 1–66 were amplified by PCR using the following primer pairs: cggaattcagattatcgtccaaaagtgttaatgc/cgaattcctagaccaagctttggatttcatt (CD95 wild type), cggaattctgccataagccctgtcctccaggt/cgaattcctagaccaagctttgtatttcatt (CD95^(Δ1–42)^), cggaattcgtgccctgccaagaagggaaggag/cgaattcctagaccaagctttgtatttcatt (CD95^(Δ1–66)^). PCR products were digested with EcoRI and inserted into an EcoRI-linearized pcDNA3-SP^CD95^HA vector. To generate the CD95 construct devoid of the death domain, a premature stop codon was inserted at amino acid position 210 using the primers cgaaaacaattgagattatcgtccaaaagtgttaatgc/cggaattctcaatcagataaatttattgccactgtttc. Next, the amplicons were digested with MfeI and EcoRI and inserted in an EcoRI-linearized pcDNA3-PS^CD95^HA vector. The chimeric CD95-gp130 construct corresponds to the ectodomain of CD95 fused to the transmembrane and intracellular regions of the IL-6 gp130 receptor. Extracellular CD95 was isolated from the pEDr-wild-type CD95 plasmid by XhoI/BamHI digestion and sub-cloned into a XhoI/BamHI-cleaved pBluescript SK vector. Extracellular CD95 was digested by XhoI/XbaI [15]. Using site-directed mutagenesis, a XbaI site was created six nucleotides upstream of the transmembrane domain of gp130 [16]. The pEDr vector encoding gp130 was then cleaved using XhoI/XbaI digestion and the gp130 ectodomain was replaced with the extracellular domain of CD95. CD95(1–42)-, CD95(1–66)- and CD95(1–158)-mCherry constructs were generated by amplifying the fragments 1–42, 1–66 and 1–158 of CD95 using the primer pairs cggaattcctgggcatctggaccctccta/ccgctcgaggaattggccatcatgatgcaggcc, cggaattcctgggcatctggaccctccta/ccgctcgaggcagtctggttcatccccattg and cggaattcctgggcatctggaccctccta/ccgctcgagcaagttagatctggatccttcctc, respectively. PCR products were digested by EcoRI/XhoI and inserted into an EcoRI/XhoI-linearized pcDNA3-(SP^CD95^HA) vector. Next, mCherry was amplified from the pmCherry-1 Vector (Clontech) using the primer pair gctctagagtgagcaagggcgaggaggac/gctctagattacttgtacagctcgtccatgccg and the amplicon was digested by XbaI and inserted in the reading frame with the extracellular regions of CD95. To generate secreted mCherry, the mCherry sequence was amplified using the primers cgggatccgtgagcaagggcgaggaggataac/ccgctcgagttagaattccttgtacagctcgtccatgc, digested by BamHI/EcoRI and inserted into the homemade vector pcDNA3-(SP^CD95^Flag) encompassing the SP of CD95 fused with the Flag tag. Each sequence was verified by sequencing (GATC, Mulhouse, France).

### Cell Death Assays

Cell viability was assessed by quantifying either the metabolic activity (MTT assay) or the cell morphology (flow cytometry), exactly as previously described [17], [18]. In brief, 4.10^4^ cells were cultured for 16 hours in flat-bottom, 96-well plates with the indicated concentrations of the apoptosis inducer in a final volume of 100 µl. 15 µl of MTT (5 mg/ml in PBS) solution was added, and after 4 hours of incubation at 37°C, the absorbance was measured at 570 nm wavelength using the Titertek Labsystems Multiskan reader (Turku, Finland).

### Flow Cytometry Analysis

All steps were performed at 4°C. Cell membranes were saturated with PBS/1% (w/v) BSA, washed with PBS and stained with anti-CD95 mAb (clone DX2) or anti-HA mAb (clone HA.11) for 30 min at 4°C. Cells were incubated for 30 min with a FITC-conjugated secondary antibody and immediately analyzed using a FACSCalibur (BD Bioscience).

### Immunoblot Analysis

Cells were lyzed for 30 minutes at 4°C in lysis buffer (25 mM HEPES pH 7.4, 1% v/v Triton X-100, 150 mM NaCl, 2 mM EGTA supplemented with a mix of protease inhibitors (Sigma-Aldrich)). Protein concentration was determined by the bicinchoninic acid method (PIERCE, Rockford, IL, USA) according to the manufacturer's protocol. Proteins were separated on 10 or 12% SDS-PAGE and transferred to a nitrocellulose membrane (GE Healthcare, Buckinghamshire, England). The membrane was blocked 15 minutes with TBST (50 mM Tris, 160 mM NaCl, 0.05% v/v Tween 20, pH 7.8) containing 5% w/v dried skimmed milk (TBSTM). Primary antibody was incubated overnight at 4°C in TBSTM. The membrane was intensively washed (TBST) and then the peroxydase-labeled anti-rabbit (Zymed Laboratories, Inc, San Francisco, CA, USA) or anti-mouse (GE Healthcare) was added for 45 minutes. The proteins were visualized with the enhanced chemiluminescence substrate kit (ECL, GE Healthcare).

### Gel Filtration

CD95-mCherry constructs were secreted after calcium/phosphate-based transfection of HEK cells in serum-deprived Opti-MEM (Life Technologies, Saint Aubin, France). Supernatants were concentrated using centricon filters and resolved in Sephacryl S-300 and S-200 High Resolution columns (GE Healthcare) equilibrated with PBS. Using an ÄKTAprime plus apparatus (GE Healthcare), proteins were eluted at a flow rate of 0.5 mL/min and 60 fractions of 2 ml were harvested and analyzed by immunoblot analysis with anti-DsRed mAb.

## Results and Discussion

**Figure 1:**
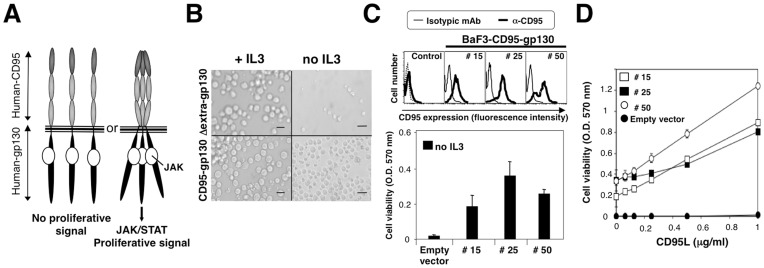
A chimeric CD95-gp130 construct confirms the presence of a PLAD in the CD95 ectodomain. A. The intracellular domain of gp130 is pre-associated with JAK kinase. If the ectodomains of CD95 are pre-associated, the intracellular regions of a CD95-gp130 construct will be brought in close proximity, inducing the trans-activation of JAKs and the implementation of a pro-survival signal. **B.** IL3-addicted BaF3 cells were transfected with ΔExtra-gp130 or CD95-gp130. At 24 hours after transfection, living cells were incubated for 5 days with or without IL3. For each condition, pictures were taken at 10x magnification. Images are representative of five pictures taken in different fields. **C.** BaF3 cells were transfected with CD95-gp130 or empty vector (numbering indicates different clones). Stable clones were selected and the expression of CD95-gp130 was assessed by flow cytometry. MTT assay was used to assess the cell viability of BaF3 cells in medium deprived of IL3. **D.** BaF3 cells were treated or untreated with the indicated concentrations of cleaved CD95L in IL3-deprived medium and cell viability was quantified using MTT viability assay.

Initially described as the interleukin-6 (IL6) receptor, gp130 is a transmembrane receptor capable of activation by a number of cytokines, including IL6, IL11, leukemia inhibitory factor (LIF), oncostatin M (OSM), and ciliary neurotrophic factor (CNTF) (for review see [19]). A key feature of this receptor is that its intracellular domain is pre-associated with Janus kinases (*i.e.,* Jak1, Jak2 and Tyk2), whose close proximity is required for activation by trans-phosphorylation and the induction of STAT1/3 (signal transducer and activator of Transcription)-mediated pro-proliferative and survival pathways [20]. We postulated that if the extracellular region of CD95 contains a homotypic interaction motif that promotes pre-association of CD95 in a ligand-independent manner, fusion of this ectodomain to the intracellular region of gp130 would elicit a pro-survival signal in the absence of CD95L binding. BaF3 cells constitute an ideal model system in which to test this hypothesis, as they rely on IL3-mediated JAK/STAT signaling for survival and proliferation [12]. These cells were transiently transfected to express either gp130 devoid of the extracellular domain (ΔExtra-gp130) [12] or the ectodomain of CD95 fused to the transmembrane and intracellular regions of gp130 (CD95-gp130) (Fig. 1A). As shown in Figure 1B, when maintained in IL-3 deprived medium, no living cells expressing ΔExtra-gp130 were detected. However, a population of CD95-gp130 transfected BaF3 cells survived despite the absence of IL3. To further confirm that the survival signal occurred through CD95-gp130 expression, stable BaF3 clones expressing CD95-gp130 were generated (Fig. 1C). In agreement with the results observed following transient expression, BaF3 cells expressing CD95-gp130 exhibited a basal proliferative rate in IL3-deprivated medium, while all empty vector-transfected BaF3 cells died when cultured in the absence of IL3. In addition, exposure to homotrimeric CD95L (metalloprotease-cleaved CD95L, described in [11]) significantly enhanced the viability of CD95-gp130-expressing cells in a dose-dependent manner, while it failed to enhance the survival of control cells (Fig. 1D). These findings confirmed that the ectodomain of CD95 contains a homotypic interaction domain necessary and sufficient to promote pre-association of the death receptor (at least two molecules) and that the magnitude of aggregation is increased and/or conformation is altered upon binding of CD95L to CD95.

**Figure 2:**
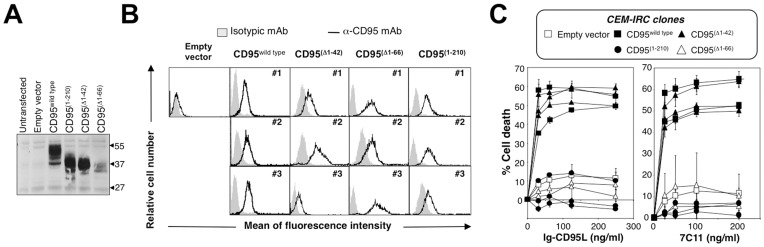
The PLAD is instrumental in transmission of the apoptotic signal and does not encompass residues 1 to 42 of CD95. A. CD95-deficient CEM-IRC cells were transfected with the indicated cDNA and living cells were isolated by Ficoll gradient and lysed. For each condition, 100 µg of protein was loaded per lane and immunoblot analysis was performed using an anti-CD95 mAb (C20). Data are representative of three independent experiments. **B.** Three independent CEM-IRC clones stably expressing CD95^wild type^, CD95^(Δ1–42)^, CD95^(Δ1–66)^ CD95^(1–210)^ were selected and the amount of CD95 construct at the plasma membrane was analyzed by flow cytometry. **C.** CEM-IRC cells shown in **F** were incubated for 24 hours with the indicated concentrations of Ig-CD95L (left panel) or 7C11 (right panel). Cell death was assessed by MTT assay. Results are given as the means ± SD of three independently performed experiments.

To identify the minimal region involved in CD95 self-aggregation, we next examined the effect of wild-type and various PLAD deletion mutants of CD95 [7] in a T-cell line expressing a faint background of endogenous CD95 (CEM-IRC, see Fig. 2A & 2B). Wild-type CD95 (CD95^wild type^) and mutants corresponding to the truncation of amino acids 1 to 42 (CD95^(Δ1–42)^), 1 to 66 (CD95^(Δ1–66)^) or the intracellular DD (CD95^(1–210)^) were all expressed at the expected molecular weight in the CEM-IRC cells, as shown in Figure 2A. We next selected stable CEM-IRC clones expressing CD95^wild type^, CD95^(Δ1–42)^, CD95^(Δ1–66)^ or CD95^(1–210)^ (Fig. 2B) and analyzed CD95-mediated apoptosis. While the expression of wild-type CD95 restored CD95-mediated apoptotic signaling in CEM-IRC cells exposed to Ig-CD95L or the agonistic anti-CD95 antibody 7C11 (Fig. 2C), mutants that lacked the DD or the complete CRD1 (CD95^(1–210)^ and CD95^(Δ1–66)^, respectively) failed to transmit cell death signal (Fig. 2C). Importantly, our data revealed that in a CD95-deficient background, elimination of the first 42 amino acids of CD95 did not affect the induction of apoptosis (Fig. 2C), contradicting previous findings showing that this domain is mandatory for the pre-association of CD95 and thus for transmission of the apoptotic signal [7], [9]. It is worth noting that deleting the entire CRD1 (amino acids 1 to 66) did not abolish 7C11 or CD95L binding ruling out the possibility that CD95^(Δ1–66)^ failed to transmit apoptotic signaling due to a loss of its interaction with apoptosis inducers (data not shown). These findings demonstrate that the first 42 amino acids of CD95 are not crucial for CD95 function and are therefore unlikely to contribute to its pre-association.

**Figure 3:**
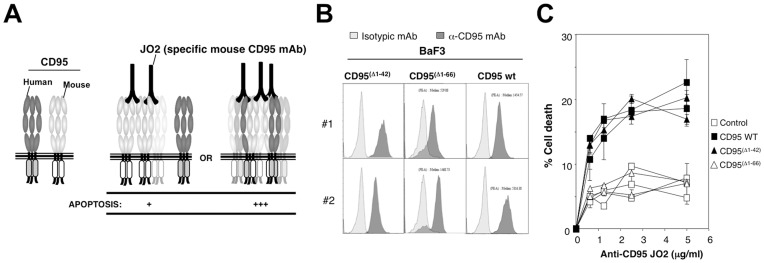
Another cellular model confirms that the PLAD does not encompass residues 1 to 42 of CD95. A . The anti-mouse CD95 mAb, clone Jo2, induces cell death by aggregating mouse CD95. If human CD95 forms a supernumerary and functional pre-associated hetero-complex with its mouse ortholog, the Jo2-mediated apoptotic signal will be enhanced. **B.** For each construct, two independent BaF3 clones were selected and the amount of human CD95 at the cell surface was assessed by flow cytometry. **C.** The BaF3 cells depicted in **B** were incubated for 24 hours with the indicated concentration of Jo2 and cell death was assessed by MTT assay. Results are given as the means ± SD of three independently performed experiments.

To further investigate the role of amino acids 1–42 in CD95 auto-aggregation, we developed a second cellular model. Because mouse and human CD95 exhibit 49% sequence identity, we hypothesized that the human CD95 PLAD could interact with its murine counterpart to assemble trimeric receptors composed of both human and mouse subunits. Accordingly, the apoptotic signal induced by the agonistic anti-CD95 mAb JO2, which is only able to bind and aggregate the mouse receptor, should be enhanced if human CD95 associates with endogenous mouse CD95 and forms a functional heterozygous complex (Fig. 3A). Human CD95^wild type^ and CD95^(Δ1–42)^, CD95^(Δ1–66)^ mutants were transfected into BaF3 cells and stable clones were isolated (Fig. 3B). BaF3 cells express a minimal amount of endogenous mouse CD95 (data not shown) and exhibit only weak sensitivity to JO2 (Fig. 3C). Ectopic expression of human wild-type CD95 enhanced the JO2-induced apoptotic signal, confirming that human and mouse CD95 can form functional heteromultimers (Fig. 3C). By contrast, expression of CD95^(Δ1–66)^ did not modify the JO2-triggered apoptotic signal, indicating that despite a propensity to auto-aggregate [21], the intracellular DD of CD95 is not sufficient to form oligomers with mouse CD95. In addition, it demonstrated that CRD1 encompasses a homotypic region that plays a pivotal role in pre-association of CD95. Confirming our previous data and similar to wild-type CD95, human CD95^(Δ1–42)^ significantly enhanced the JO2-driven apoptotic signal (Fig. 3C). This result indicates that the region between amino acids 43 and 66 is instrumental in oligomerization of CD95.

**Figure 4:**
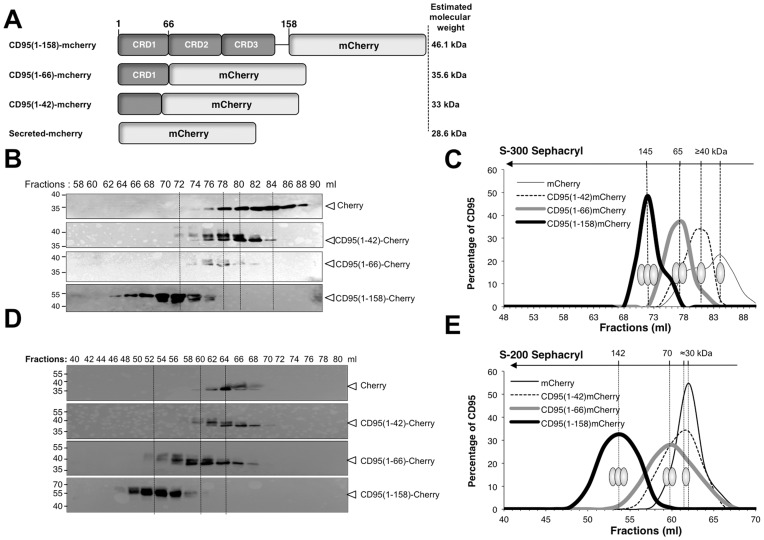
The CD95 self-association domain covers the residues 43 to 66. A. Schematic representation of the different CD95-mCherry constructs. **B.** Supernatants from HEK cells transfected with the constructs depicted in **A** were fractionated by gel filtration using a S-300 HR column. Seventeen fractions were harvested and analyzed by immunoblot analysis (anti-DsRed). **C.** Densitometry analyses were performed on the immunoblots shown in **B** using Fiji software. For each construct, the estimated quaternary structure is depicted. **D.** Supernatants from HEK cells transfected with the constructs depicted in **A** were fractionated by gel filtration using a S-200 HR column. Twenty-one fractions were harvested and analyzed by immunoblot analysis (anti-DsRed). **E.** Densitometry analyses were performed on the immunoblots shown in **D** using Fiji software. For each construct, the estimated quaternary structure is depicted.

To confirm that the first 42 residues are not mandatory for pre-association of the death receptor, we fused the full-length ectodomain of CD95 (CD95(1–158)-mCherry), the complete CRD1 region (CD95(1–66)-mCherry) or amino acids 1 to 42 (CD95(1–42)-mCherry) to a monomeric carrier (fluorescent protein mCherry; Fig. 4A) and the stoichiometry of each chimeric protein was assessed by size-exclusion chromatography. After transfection, HEK supernatants containing the different constructs were fractionated in two gel filtration columns covering a resolution range for intermediate- (Sephacryl-300 HR with a fractionation range comprised between 1×10^4^ and 1.5×10^6^ daltons) (Fig. 4B & 4C) or low-molecular weight proteins (Sephacryl-200 HR with a fractionation range comprised between 5×10^3^ and 2.5×10^5^ daltons) (Fig. 4D & 4E). The estimated molecular mass of the monomer for each recombinant protein is depicted in Figure 4A. The complex formed by the ectodomain of CD95 fused to mCherry was eluted from the two columns as a homotrimer (≈145 kDa) (Fig. 4B–C–D–E). By contrast, mCherry alone and CD95(1–42)-mCherry were resolved in fractions corresponding to molecular weights lower than 40 kDa (Fig. 4B–C–D–E), confirming that the first 42 amino acids are not sufficient to mediate auto-aggregation of the receptor. On the other hand, CD95(1–66)-mCherry failed to form homotrimers but aggregated as homodimer (Fig. 4B–C–D–E) since the protein was mainly detected in fractions covering 65 to 70 kDa depending on the gel filtration column used to resolve the native molecular masse of the chimera. Overall, these data not only validate that the stretch of amino acids 43 to 66 constitutes a CD95 homotypic interaction domain, but also reveal that the region between amino acids 67 and 158 favors the formation of CD95 quaternary structure.

Discrepancy concerning the inclusion of the first 42 amino acids in the PLAD may be explained by the fact that the CD95 constructs used by Ruberti’s group were generated via *in vitro* translation or prokaryotic systems, neither of which are capable of glycosylation [9]. The extracellular domain of CD95 contains two putative glycosylation sites in the first 42 amino acids: an *N*-glycosylation at Asp 32 [22] and a threonine-rich motif (Thr24 to Thr30) that resembles *O*-glycosylated mucin-type sequences [23]. In agreement with previous reports showing that sialylation of CD95 inhibited apoptotic signaling [24], [25], a more recent study demonstrated that CD95 glycosylation impairs receptor oligomerization [22]. Accordingly, glycosylation of the CD95 amino-terminal domain may inhibit the potential homotypic interaction of the 1–42 region that was observed in unglycosylated CD95 mutants. It is noteworthy that although the chimeric CD95(1–66)-mCherry protein contains the necessary PLAD, it fails to form homotrimers, but rather forms homodimeric structures, suggesting that in addition to the DD, a motif located between amino acids 67 to 158 promotes auto-aggregation of CD95, or that truncation of the protein interferes with the ternary structure of CD95 and reduces homotypic affinity. Using transient transfection of T-cell lines expressing high amounts of wild-type CD95, Siegel *et al*. showed that in contrast to a DD-truncated mutant, expression of mutant in which both the first 42 amino acids and the DD (CD95-(43–210)) were deleted did not affect CD95-mediated apoptotic signaling. This indicates that without this amino-terminal region, the CD95 mutant fails to form a heterotrimer with its wild-type counterpart and thus cannot act as a dominant-negative subunit [7]. Based on our findings, although this CD95-(43–210) construct still encompasses the major homotypic interaction domain (amino acids 43 to 66), a decrease in its homotypic affinity may impair its ability to interfere with the high amounts of endogenous wild-type CD95; thus, it fails to alter the formation of wild-type CD95 homotrimers and transmission of the apoptotic signal.

Mutations in the extracellular region of CD95 represent less than 30 percent of all mutations found in ALPS type Ia patients and these mutations exhibit a lower clinical penetrance compared to intracellular mutations. Nonetheless, two mutations in the ectodomain of CD95 (C57X and D62frameshift), which abrogate the CD95-mediated apoptotic signal despite showing no affinity for the ligand, have been described [5]. These rare mutations shed light on the minimal extracellular region of CD95 necessary for auto-aggregation of the receptor. In light of recent observations showing that CD95 participates in chronic inflammation and tumor dissemination through non-apoptotic signaling pathways [11], [26]–[29], inhibiting CD95 oligomerization by interfering with the PLAD may represent a novel therapeutic strategy able to hamper glioma metastasis or dampen crisis in lupus patients [11], [29].
